# The Detection of Influenza Virus Before and During the COVID‐19 Pandemic in Cameroon

**DOI:** 10.1111/irv.13313

**Published:** 2024-05-17

**Authors:** Gwladys Chavely Monamele, Desmon Toutou Tsafack, Chanceline Ndongo Bilounga, Mohamadou Njankouo Ripa, Christian Nsangou Yogne, Hermann Landry Munshili Njifon, Felix Nkom, Ubald Tamoufe, Linda Esso, Fancioli Koro Koro, Ronald Perraut, Richard Njouom

**Affiliations:** ^1^ Virology Service Centre Pasteur of Cameroon Yaounde Cameroon; ^2^ Faculty of Health Sciences University of Buea Buea Cameroon; ^3^ Department of Biochemistry University of Douala Douala Cameroon; ^4^ Department for the Control of Diseases, Epidemics and Pandemics (DLMEP) Ministry of Public Health Yaounde Cameroon; ^5^ Garoua Annex Centre Pasteur of Cameroon Garoua Cameroon; ^6^ Metabiota, Inc Yaounde Cameroon

**Keywords:** Cameroon, COVID‐19 pandemic, epidemiology, influenza, SARS‐CoV‐2

## Abstract

**Background:**

Influenza and severe acute respiratory syndrome coronavirus 2 (SARS‐CoV‐2) are both respiratory viruses with similar clinical manifestations and modes of transmission. This study describes influenza data before and during the coronavirus disease pandemic (COVID‐19) in Cameroon and SARS‐CoV‐2 data during the pandemic period.

**Methods:**

The study ran from 2017 to 2022, and data were divided into two periods: before (2017–2019) and during (2020–2022) the COVID‐19 pandemic. Nasopharyngeal samples collected from persons with respiratory illness were tested for influenza using the Centers for Disease Control and Prevention (CDC) typing and subtyping assays. During the COVID‐19 pandemic, the respiratory specimens were simultaneously tested for SARS‐CoV‐2 using the *DaAn* gene protocol or the Abbott real‐time SARS‐CoV‐2 assay. The WHO average curve method was used to compare influenza virus seasonality before and during the pandemic.

**Results:**

A total of 6246 samples were tested. Influenza virus detection rates were significantly higher in the pre‐pandemic period compared to the pandemic period (30.8% vs. 15.5%; *p* < 0.001). Meanwhile, the SARS‐CoV‐2 detection rate was 2.5%. A change in the seasonality of influenza viruses was observed from a bi‐annual peak before the pandemic to no clear seasonal pattern during the pandemic. The age groups 2–4 and 5–14 years were significantly associated with higher influenza positivity rates in both pre‐pandemic and pandemic periods. For SARS‐CoV‐2, all age groups above 15 years were the most affected population.

**Conclusion:**

The COVID‐19 pandemic had a significant impact on the seasonal influenza by changing the seasonality of the virus and reducing its detection rates.

## 1 Background

Seasonal influenza is an acute respiratory infection caused by influenza A and B viruses circulating in all parts of the world. Influenza viruses belong to the family Orthomyxoviridae and represent a year‐round disease burden. The global annual attack rate is estimated to be 5%–10% in adults and 20%–30% in children, resulting in approximately 290,000 to 650,000 deaths [1]. Because of its high burden in both developed and developing countries, influenza surveillance systems have been established by several countries worldwide. The Global Influenza Surveillance and Response System (GISRS), first implemented in 1952, has expanded from 25 countries reporting influenza data to over 127 countries [2]. With the emergence of the coronavirus disease (COVID‐19) pandemic, the sentinel surveillance systems for influenza have been used to integrate testing for severe acute respiratory syndrome coronavirus 2 (SARS‐CoV‐2) in specimens collected from influenza surveillance sources [3].

SARS‐CoV‐2, a member of the Coronaviridae family, was first identified in Wuhan in December 2019 and has caused significant morbidity with more than 600 million infections in 4 years and approximately 7 million deaths worldwide [4]. SARS‐CoV‐2 mortality rates during the pandemic were at least three times higher than influenza mortality rates. In Cameroon, the first case of COVID‐19 was recorded in March 2020, and by April 2023, 124,983 cases and 1971 deaths had been recorded [5]. Influenza and SARS‐CoV‐2 have similar clinical presentations, including but not limited to fever, cough, malaise, dyspnoea, runny nose, sore throat, headache and myalgia [6, 7]. Therefore, only laboratory diagnosis can confirm infection with either respiratory virus.

Since 2009, a National Influenza Centre (NIC) has been established at the Centre Pasteur Cameroon to monitor respiratory pathogens, including influenza virus and SARS‐CoV‐2. Prior to the COVID‐19 pandemic, reports showed year‐round influenza circulation with a bimodal seasonal pattern: a major peak between September and December and a minor peak between March and June [8, 9]. As influenza virus and SARS‐CoV‐2 share similar modes of transmission and preventive measures, the COVID‐19 pandemic may have altered the frequency of influenza virus circulation and affected its annual distribution. In this manuscript, we perform a comparative analysis of influenza data before (2017–2019) and during the COVID‐19 pandemic (2020–2022) in terms of sociodemographic and clinical characteristics of affected populations and influenza virus seasonality. We also describe SARS‐CoV‐2 data collected during the period of co‐circulation of both viruses.

## 2 Methods

### 2.1 Study Sites

**FIGURE 1:**
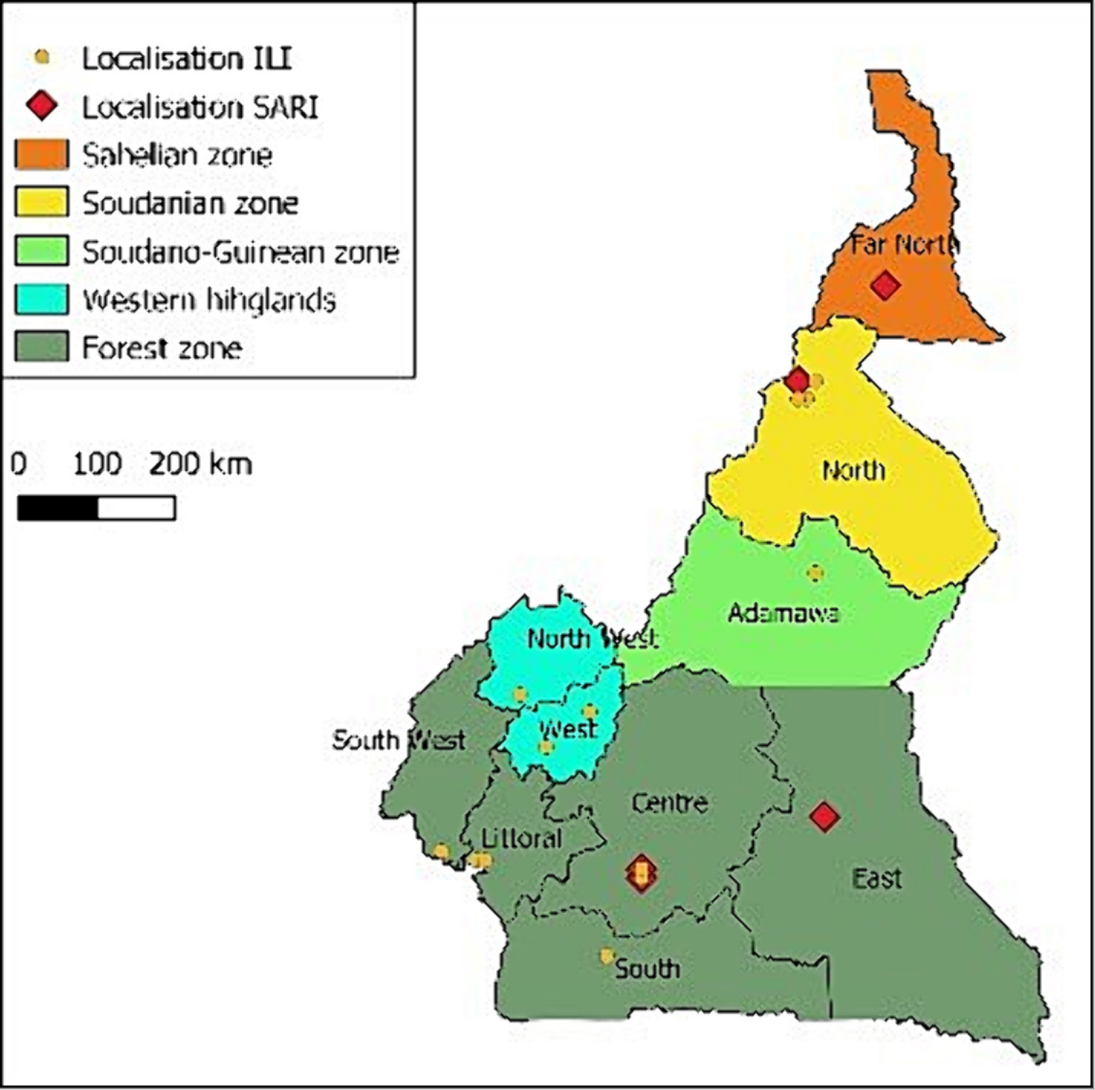
Map showing location of sentinel sites.

For 15 years, the Centre Pasteur of Cameroon (CPC) has been designated as the NIC in Cameroon. This surveillance system originally focused on influenza virus but now includes surveillance for other respiratory pathogens and SARS‐CoV‐2. Currently, all 10 regions of Cameroon have at least one sentinel site (Figure 1). Some sites are referred to as influenza‐like illness (ILI) sites because they collect specimens from persons suspected of having ILI, whereas others are referred to as severe acute respiratory illness (SARI) sites because they collect specimens from persons with SARI requiring hospitalisation. There are five sites in the centre region, two of which are SARI sites: *Centre d'Animation Social et Sanitaire de Nkolndongo*; *Centre Médico‐Social de l'Ambassade de France*, *Centre Médical Marie Reines d'Etoudi*, *Centre Hopitalier d'Essos* (SARI) and Jamot Hospital in Yaoundé (SARI). The northern region has three ILI sites and one SARI site: *CSI de Poumpoure*, *CSI de Foulbéré*, *CSI de Roumde Adjia* and Garoua Regional Hospital (SARI). The west and littoral regions each have two sentinel sites each: *CSI de Bandjoun* (west), *CSI de Kueka* (west), *Hôpital Catholique Saint Albert le Grand* (littoral) and *Hôpital Catholique Notre Dame de l'Amour* (littoral). There is one ILI site in each of the remaining regions: Bertoua Regional Hospital (east), Ebolowa Regional Hospital (south), Mount Mary Hospital Buea (south west), Saint Blaise Catholic Hospital Big Mankon (north west), Hôpital Regional de Maroua (far north) and *CSI de Marza* (Adamawa).

### 2.2 Specimen Collection

From 2017 to 2022, respiratory specimens were collected from individuals who met the World Health Organization (WHO) case definition of ILI or SARI [10]. ILI was defined by an acute respiratory infection with fever and cough, within 10 days of symptom onset, whereas SARI was defined by patients presenting with fever (or history of fever) and cough with onset within the previous 10 days and requiring hospitalisation. The sample consisted of nasopharyngeal or oropharyngeal swabs collected from the suspected cases and placed in 2 mL cryovials containing viral transport medium. All samples were transported to the CPC where they were processed and analysed according to WHO standard procedures [10].

### 2.3 RNA Extraction

The QIAamp Viral RNA Mini Kit (Qiagen, Hilden, Germany) was used to extract RNA from 140 μL of clinical samples into a final volume of 60 μL elution buffer according to the manufacturer's instructions. After nucleic acid extraction, all samples were tested for influenza virus and SARS‐CoV‐2.

### 2.4 Detection of Influenza Types and Subtypes

Extracts were tested for the presence of influenza using the Centers for Disease Control and Prevention (CDC) influenza A/B typing assay in an ABI Prism 7500 thermocycler (Applied Biosystems, Foster City, California, USA). Positive samples were then subtyped using the CDC subtyping kits. All polymerase chain reaction (PCR) reactions for influenza virus detection were performed using an enzyme‐based Invitrogen SuperScript™ III Platinum one‐step quantitative reverse transcription‐polymerase chain reaction (RT‐PCR) system (Thermo Fisher Scientific, Massachusetts, USA). The reaction mixture for the detection of all influenza virus types and subtypes was composed as follows: 12.5 μL of 2X PCR Master Mix, 0.5 μL of Reverse Transcriptase/Taq polymerase, 0.5 μL of ROX (carboxy‐X‐rhodamine), 2 μL of 10 μM forward and reverse primers, 2 μL of 2.5 μM probe, and 5 μL RNA extract. All primers and probes were designed for universal detection of influenza A and B viruses and all subtyping primers and probes for the specific detection of A/H3, A/H1(2009), B/Yamagata and B/Victoria viruses. The following cycling conditions were used: one cycle of reverse transcription (30 min at 50°C), followed by inactivation of Taq polymerase inhibitors at 95°C for 2 min, and 45 cycles of amplification at 95°C for 15 s and 55°C for 30 s. Samples were considered positive for influenza if the threshold cycles (Ct) were less than 37.

### 2.5 Detection of SARS‐CoV‐2 Viruses

During the COVID‐19 pandemic, SARS‐CoV‐2 diagnosis was performed using the *DaAn* Gene protocol (Daan Gene Co., Ltd., of Sun Yat‐sen University, China; EUL 0493‐141‐00), which targets the open reading frame (ORF) 1ab and nucleocapsid (N) genes, or the *Abbott* RealTime SARS‐CoV‐2 Assay (Abbott Molecular Inc., USA, EUL‐0503‐027‐00), which targets the RNA‐dependent RNA polymerase (RdRp) and N genes [11].

The *DaAn* gene protocol has a lower limit of detection of 500 copies/mL. This assay used RNA extracted with the QIAamp Viral RNA Mini Kit as described previously. The master mix for this assay consisted of ready‐to‐use solutions containing specific primers, probes, buffer, Taq DNA polymerase and c‐MMLV reverse transcriptase enzymes. The amplification reaction for the DaAn gene protocol was as follows: reverse transcription (50°C for 15 min), Taq polymerase activation (95°C for 15 min), 45 cycles of two‐step denaturation (94°C for 15 s) and hybridisation (55°C for 45 s).

Meanwhile, for SARS‐CoV‐2 detection using the *Abbott* rRT‐PCR assay, 500 μL of each sample was first added to DNase‐RNase‐free water heated at 70°C for 10 min. The inactivated sample was loaded into the *Abbott* m2000sp instrument for automated extraction. Amplification was then performed on the m2000rt thermocycler using the master mix preparation containing an internal control, primers and probes. This assay has a detection sensitivity of 100 copies/mL.

Samples were considered positive for SARS‐CoV‐2 if threshold cycles (Ct) were less than 37 were obtained for both assays. As integrated surveillance for SARS‐CoV‐2 started in mid‐June 2021, samples collected prior to that date were retrospectively tested for SARS‐CoV‐2 using the *DaAn* Gene protocol.

### 2.6 Ethical Statement

Approval‐to‐use data collected from the routine influenza surveillance system for research purposes was obtained from the University of Douala Institutional Ethics Committee for Research on Human Health (No. 3971/CEI‐Udo/07/2023/M) and the Cameroon National Ethics Committee (Reference No. 2016/08/798/CE/CNERSH). The identity of participants from the sentinel sites was coded to ensure confidentiality of the data collected. Names were not disclosed but were written in the format *AAA BBB*, where AAA and BBB correspond to the first three letters of the participant's surname and first name, respectively. Once in the laboratory, each sample was assigned a unique identifier, which was used at all stages of sample analysis. Informed written consent was obtained from the study participants and/or their legal guardians prior to sample collection.

### 2.7 Data Analysis

Individual data were entered into a Microsoft Access database (Microsoft, Washington, DC, USA) using a pre‐designed questionnaire containing information on participant identification, demographics and clinical status. This information was then retrieved, and statistical analysis was performed using SPSS software Version 22.0, and figures were generated using Microsoft 365 Excel software (Microsoft, Washington, DC, USA). Binary logistic regression was used to determine associations between influenza virus detection rates before and during the COVID‐19 pandemic and between the detected viruses and socio‐demographic or clinical data. For each characteristic of interest, a reference group was selected for comparisons to calculate the odds of infection. *P* values less than 0.05 were considered statistically significant. The WHO average curve method (https://worldhealthorg.shinyapps.io/averagecurves/) was used to determine the seasonality of influenza virus in Cameroon prior to the COVID‐19 pandemic. Data collected from 2017 to 2019 were used as historical data to establish the epidemic and intensity thresholds, which were compared with influenza seasons during the pandemic period (2020–2022). The epidemic threshold was obtained by calculating the median historical rate. Intensity thresholds for the average curve were calculated from the historical peaks, and the mean and standard deviation of their intensities. The 40%, 90% and 97.5% point intervals were used to calculate the moderate, high and extraordinary thresholds, respectively, using the normal distribution.

## 3 Results

### 3.1 Description of Study Population

During the study period, a total of 6246 samples were tested: 3661 (58.6%) were collected during the pre‐pandemic period and 2585 (41.4%) during the COVID‐19 pandemic. Of these, 2805 (50.2%) were male, and 2782 (49.8%) were female. The age of the participants ranged from 2 weeks to 95 years with a mean of 11.1 ± 0.2 years. The most represented age group was the 0–1‐year and the 2–4‐year age groups, which accounted for approximately 65% of the total population. Most of the participants presented with mild disease, represented here by ILI at about 78%, and a smaller proportion presented with severe disease (SARI). Nine regions of Cameroon were represented, with the centre (1955/6246; 31.3%), north (24.2%), west (17.5%) and littoral (12.0%) regions being more represented.

### 3.2 Influenza and SARS‐CoV‐2 Viruses Detected Before and During the COVID‐19 Pandemic

During the entire study period, a total of 1527 (24.4%) participants were positive for influenza virus. The influenza positivity rate was significantly higher in the pre‐pandemic period than in the pandemic period (30.8% vs. 15.5%; *p* < 0.001). Meanwhile, 64 cases of SARS‐CoV‐2 were detected during the pandemic period, representing 2.5%.

**FIGURE 2:**
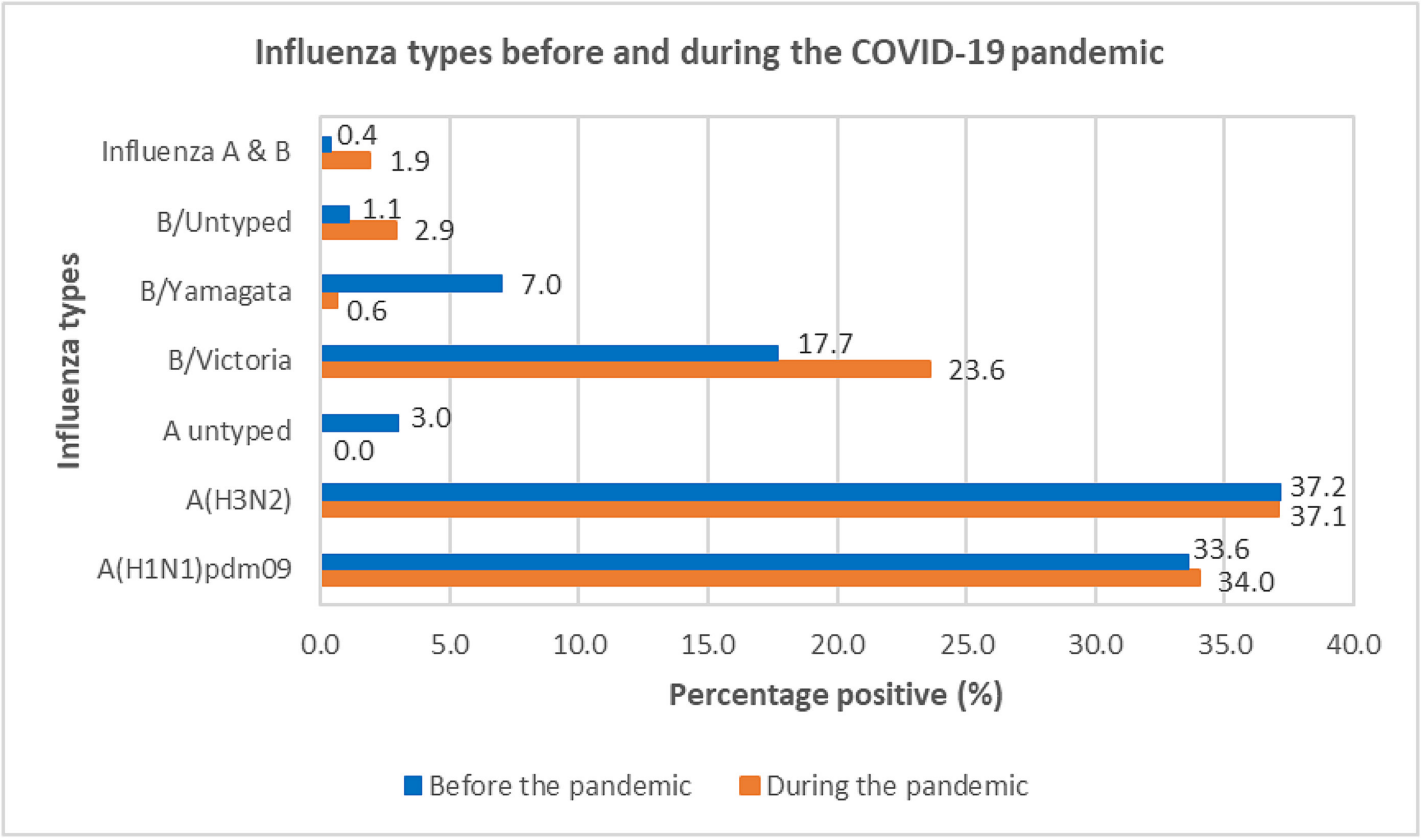
Influenza viruses detected before and during the COVID‐19 pandemic.

The distribution of influenza virus types and subtypes was similar in the pre‐pandemic and pandemic periods. The predominant virus type identified was influenza A(H3N2) (37.2% before vs. 37.1% during the pandemic), followed by A(H1N1)pdm09 (33.6% before vs. 34.0% during the pandemic) and lineage B/Victoria (17.7% before vs. 23.6% during the pandemic) and finally B/Yamagata (7.0% before vs. 0.6% during the pandemic) (Figure 2). Other viral profiles found in both periods were untyped influenza A, untyped influenza B and co‐infection of influenza A and B, which were less common. A major difference in the distribution of influenza viruses before and during the pandemic was related to the B/Yamagata lineage. During the pandemic, only three cases of the B/Yamagata lineage were detected period in the month of January 2020. In addition, one case of B/Victoria lineage was co‐infected with SARS‐CoV‐2 virus.

### 3.3 Association Between Influenza and Participant Demographic and Clinical Data Before and During the Pandemic

Comparison of the socio‐demographic and clinical characteristics of participants before and during the COVID‐19 pandemic showed that more factors were associated with influenza during the pandemic (age group, illness type, duration, region) compared with the pre‐pandemic period (age group, region). The 2–4‐ and 5–14‐year age groups were the most affected population with influenza virus in both the pre‐pandemic and pandemic periods. In the pre‐pandemic period, the 15–49‐year age group was additionally associated, although with a lower odds ratio (*p* = 0.011, OR = 1.3). We also observed that the influenza virus detection rate was significantly higher in ILI (mild) cases than in SARI (severe) cases during the pandemic, whereas the proportions were similar before the pandemic. Samples collected within 5 days of illness onset had higher influenza positivity rates than those collected 6–10 and more than 10 days after illness onset. This association was significant during the pandemic period. Regarding the origin of the samples, the centre and littoral regions had the highest proportion of influenza cases in the pre‐pandemic period, whereas only the littoral region had significantly higher influenza virus detection rates during the pandemic (Table 1).

**TABLE 1:** Association between influenza and demographic and clinical parameters before and during the pandemic.

Characteristics		Before the pandemic					During the pandemic				
		Number tested	Influenza *N* (%)	*p*	OR	CI	Number tested	Influenza *N* (%)	*p*	OR	CI
Age group (years)	0–1	1257	293 (23.3)	Ref	Ref	Ref	847	115 (13.6)	Ref	Ref	Ref
	2–4	793	322 (40.6)	< 0.001*	2.2	[1.9–2.7]	639	142 (22.2)	< 0.001*	1.8	[1.4–2.4]
	5–14	452	187 (41.4)	< 0.001*	2.3	[1.8–2.9]	310	62 (20.0)	0.008*	1.6	[1.1–2.2]
	15–49	557	161 (28.9)	0.011*	1.3	[1.1–1.7]	395	50 (12.7)	0.657	0.9	[0.6–1.3]
	50–64	95	27 (28.4)	0.260	1.3	[0.8–2.1]	92	7 (7.6)	0.111	0.5	[0.2–1.2]
	≥ 65	72	14 (19.4)	0.450	0.8	[0.4–1.4]	82	6 (7.3)	0.114	0.5	[0.2–1.2]
	Missing data	435	122 (28.0)	0.048	1.3	[1.0–1.6]	187	15 (8.0)	0.040*	0.6	[0.3–0.9]
Gender	Male	1585	475 (30.0)	Ref	Ref	Ref	1220	198 (16.2)	Ref	Ref	Ref
	Female	1613	513 (31.8)	0.946	1.1	[0.9–1.3]	1169	185 (15.8)	0.788	1.0	[0.8–1.2]
	Missing data	463	138 (29.8)	0.261	1.0	[0.8–1.2]	196	18 (9.2)	0.012	0.5	[0.3–0.9]
Clinical type	ILI	2889	896 (31.0)	Ref	Ref	Ref	1978	331 (16.7)	Ref	Ref	Ref
	SARI	772	230 (29.8)	0.514	0.9	[0.8–1.1]	607	70 (11.5)	0.002*	0.6	[0.5–0.9]
Illness duration (days)	0–5	2926	913 (31.2)	Ref	Ref	Ref	2093	342 (16.3)	Ref	Ref	Ref
	6–10	323	86 (26.6)	0.091	0.8	[0.6–1.0]	250	33 (13.2)	0.202	0.8	[0.5–1.1]
	> 10	166	42 (25.3)	0.110	0.7	[0.5–1.1]	188	14 (7.4)	0.002*	0.4	[0.2–0.7]
	Missing data	246	85 (34.6)	0.278	1.2	[0.8–1.5]	54	12 (22.2)	0.253	1.5	[0.8–2.8]
Region	Adamawa	0	0	—	—	—	32	5 (15.6)	0.666	1.2	[0.5–3.2]
	Centre	1147	471 (41.1)	Ref	Ref	Ref	808	105 (13.0)	Ref	Ref	Ref
	Far north	3	1 (33.3)	0.787	0.7	[0.1–7.9]	22	0	0.998	0	0
	Littoral	410	162 (39.5)	0.583	0.9	[0.7–1.2]	340	98 (28.8)	< 0.001*	2.7	[2.0–3.7]
	North	705	212 (30.1)	< 0.001*	0.6	[0.5–0.8]	808	120 (14.9)	0.281	1.2	[0.9–1.5]
	North west	213	33 (15.5)	< 0.001*	0.3	[0.2–0.4]	35	2 (5.7)	0.220	0.4	[0.1–1.7]
	South	192	56 (29.2)	0.002*	0.6	[0.4–0.8]	0	0	—	—	—
	South west	329	73 (22.2)	< 0.001*	0.4	[0.3–0.5]	110	15 (13.6)	0.852	1.1	[0.6–1.9]
	West	662	118 (17.8)	< 0.001*	0.3	[0.2–0.4]	430	56 (13.0)	0.989	1.0	[0.7–1.4]
Total		**3661**	**1126 (30.8)**				**2585**	**401 (15.5)**			

*Note:* Significant thresholds are indicated by (*). Bold values represent the total number of samples tested and the global prevalence of SARS‐CoV2 or influenza.

### 3.4 Association Between SARS‐CoV‐2 and Participants' Demographic and Clinical Data

For SARS‐CoV‐2, the demographic and clinical factors that were significantly associated with higher detection rates were year of sample collection, age and region. SARS‐CoV‐2 detection rates were highest in 2022 compared with earlier periods of the pandemic. Also, all age groups above 15 years were the most affected population (OR: 3.4–4.3). Samples originating from the west (5.1%) and north west (11.4%) regions had the highest proportion of SARS‐CoV‐2 infections, with a statistically significant association in the north west region (*p*: 0.028). No statistically significant association were observed with sex, illness type and illness duration (Table 2).

**TABLE 2:** Association between SARS‐CoV‐2 positivity rates and some demographic and clinical parameters.

Characteristics		Number tested	SARS‐CoV‐2 *N* (%)	*p*	OR	CI
Year	2020	633	02 (0.3)	Ref	Ref	Ref
	2021	987	10 (1.0)	0.131	3.2	[0.7–14.8]
	2022	965	52 (5.4)	< 0.001*	18.0	[4.4–74.0]
Age group (years)	0–1	847	14 (1.7)	Ref	Ref	Ref
	2–4	639	6 (0.9)	0.243	0.6	[0.2–1.5]
	5–14	310	11 (3.5)	0.055	2.1	[1.0–4.9]
	15–49	395	22 (5.6)	< 0.001*	3.5	[1.8–6.9]
	50–64	92	5 (5.4)	0.021*	3.4	[1.2–9.7]
	≥ 65	82	5 (6.1)	0.011*	3.9	[1.4–11.0]
	Missing data	187	1 (0.5)	0.272	0.3	[0.0–2.4]
Gender	Male	1220	33 (2.7)	Ref	Ref	Ref
	Female	1169	29 (2.5)	0.731	0.9	[0.6–1.5]
	Missing data	196	2 (1.0)	0.176	0.4	[0.1–1.6]
Clinical type	ILI	1978	48 (2.4)	Ref	Ref	Ref
	SARI	Ref	16 (2.6)	0.772	1.1	[0.6–1.9]
Illness duration (days)	0–5	2093	53 (2.5)	Ref	Ref	Ref
	6–10	250	9 (3.6)	0.323	1.4	[0.7–3.0]
	> 10	188	1 (0.5)	0.118	0.2	[0.0–1.5]
	Missing data	54	1 (1.9)	0.754	0.7	[0.1–5.4]
Region	Adamawa	32	0	0.998	0.0	0.0
	Centre	808	29 (3.6)	Ref	Ref	Ref
	Far north	22	0	0.998	0.0	0.0
	Littoral	340	1 (0.3)	0.013*	0.1	[0.0–0.6]
	North	808	7 (0.9)	0.001*	0.2	[0.1–0.6]
	North west	35	4 (11.4)	0.028*	3.5	[1.1–10.5]
	South	0	0	—	—	—
	South west	110	1 (0.9)	0.171	0.2	[0.0–1.8]
	West	430	22 (5.1)	0.200	1.4	[0.8–2.56]
Total		**2552**	**64 (2.5)**			

*Note:* Significant thresholds are indicated by (*). Bold values represent the total number of samples tested and the global prevalence of SARS‐CoV2 or influenza.

### 3.5 Description of Participants' Symptoms Related to Influenza and SARS‐CoV‐2 Infection

Table 3 shows the proportion of symptoms reported in the study population in relation to influenza and SARS‐CoV‐2 status. Overall, there were more symptoms that were suggestive of influenza virus infection than SARS‐CoV‐2 infection. The symptoms associated with influenza virus infection during the pre‐pandemic period were different from those observed during the COVID‐19 pandemic. During the pre‐pandemic period, significantly higher odds of influenza infection were observed in the presence of arthralgia (OR: 1.9), myalgia (OR: 1.9), sore throat (OR: 1.8), headache (OR: 1.6), asthenia (OR: 1.6) and rhinorrhoea (OR: 1.4). During the pandemic, most of the symptoms were associated with lower odds of influenza virus infection, including sore throat, asthenia, headache, vomiting, shortness of breath and diarrhoea. For SARS‐CoV‐2, significantly higher odds of infection were observed in persons with sore throat (OR: 3.1).

**TABLE 3:** Proportion of symptoms according to influenza and SARS‐CoV‐2 status.

	Influenza pre‐pandemic period				Influenza during the pandemic				SARS‐CoV‐2			
	Symptom frequency	Influenza *N* (%)	*p*	OR	Symptom frequency	Influenza *N* (%)	*p*	OR	Symptom frequency	SARS‐CoV‐2 *N* (%)	*p*	OR
Fever	672	217 (32.3)	0.069	1.3	1619	266 (16.4)	0.096	1.2	1619	41 (2.5)	0.811	1.1
Cough	820	257 (31.3)	0.189	1.2	1953	310 (15.9)	0.374	1.1	1953	48 (2.5)	0.917	1.0
Rhinorrhoea	805	258 (32.0)	0.033*	1.4	1536	255 (16.6)	0.065	1.2	1536	45 (2.9)	0.075	1.6
Asthenia	577	202 (35.0)	< 0.001*	1.6	453	45 (9.9)	< 0.001*	0.6	453	6 (1.3)	0.089	0.5
Headache	327	122 (37.3)	0.001*	1.6	530	60 (11.3)	0.003*	0.6	530	11 (2.9)	0.507	0.8
Sore throat	253	103 (40.7)	< 0.001*	1.8	438	53 (12.1)	0.031*	0.7	438	24 (5.5)	< 0.001*	3.1
Myalgia	220	91 (41.4)	< 0.001*	1.9	152	19 (12.5)	0.292	0.7	152	6 (3.9)	0.234	1.7
Arthralgia	212	88 (41.5)	< 0.001*	1.9	127	14 (11.0)	0.155	0.7	127	3 (2.4)	0.933	1.0
Noisy breath	183	50 (27.3)	0.334	0.8	209	24 (11.5)	0.095	0.7	209	4 (1.9)	0.587	0.8
Shortness of breath	139	38 (27.3)	0.413	0.8	260	20 (7.7)	< 0.001*	0.4	260	9 (3.5)	0.284	1.5
Vomiting	170	60 (35.3)	0.125	1.3	149	12 (8.1)	0.011*	0.5	149	0	0.969	0
Diarrhoea	159	41 (25.8)	0.179	0.8	142	8 (5.6)	0.002*	0.3	142	1 (0.7)	0.193	0.3
Conjunctivitis	253	84 (33.2)	0.254	1.2	279	33 (11.8)	0.073	1.4	279	8 (2.9)	0.656	1.2
Ear pain	73	19 (26.0)	0.410	0.8	94	14 (14.9)	0.866	1.0	94	4 (4.3)	0.265	1.8
Skin rash	53	9 (17.0)	0.034*	0.5	45	4 (8.9)	0.223	0.5	45	0	0.998	0

*Note:* Significant thresholds are indicated by (*).

### 3.6 Influenza Virus Seasonality Before and During the COVID‐19 Pandemic

**FIGURE 3:**
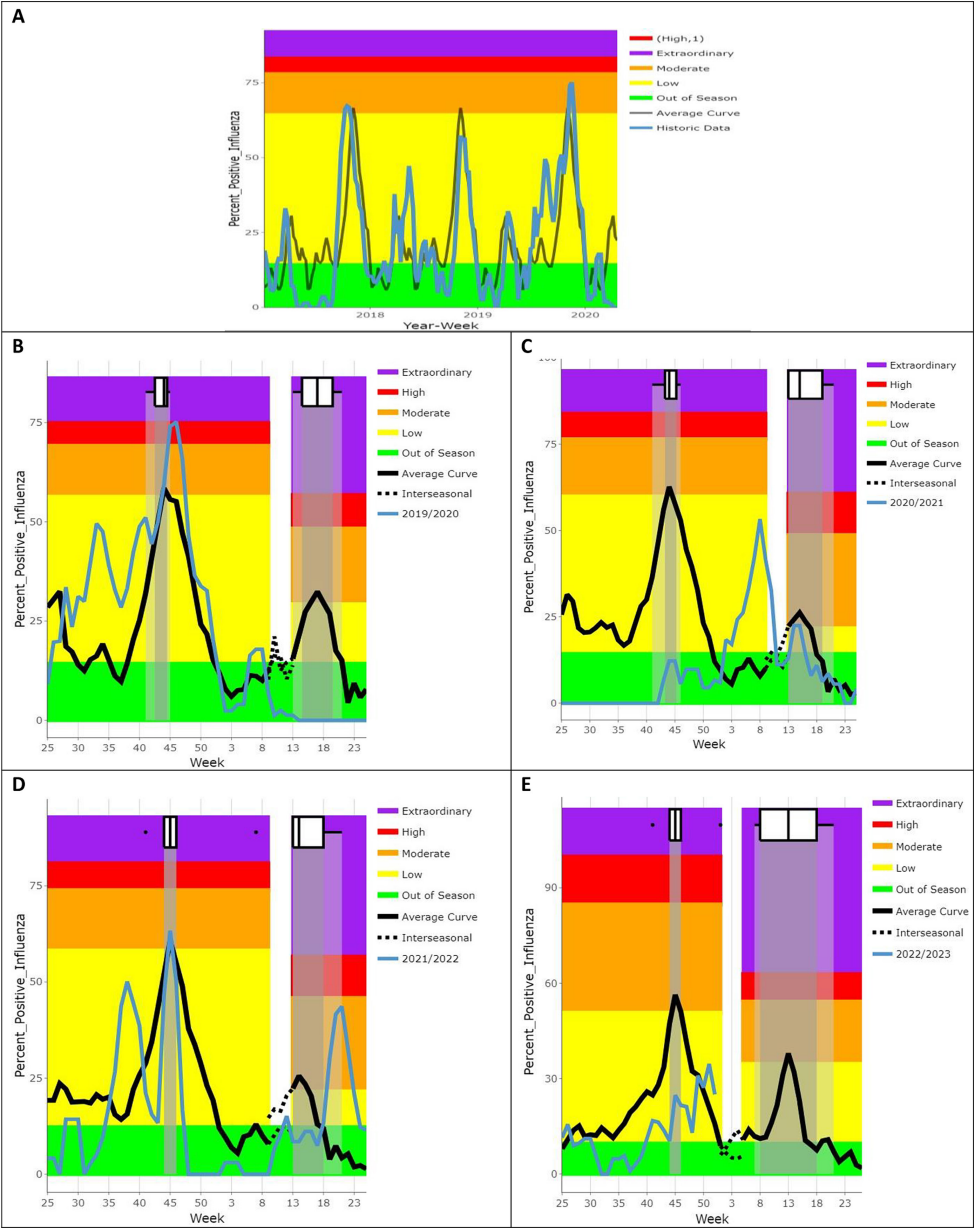
Average curves of influenza seasons before and during the COVID‐19 pandemic in Cameroon. (A) Time series of influenza modelled with the WHO average curve method for the 2017–2019 seasons. (B) 2019–2020 influenza season against modelled average curves. (C) 2020–2021 influenza season against modelled average curves. (D) 2021–2022 influenza season against modelled average curves. (E) 2022 influenza season against modelled average curves.

Using the WHO average curve method, a two‐wave model was generated for the 2017–2019 historical data from Cameroon, providing separate epidemic and intensity thresholds for influenza activity within this period (Figure 3). The epidemic threshold in Cameroon was calculated to be 14.3%. The average curve model identified a minor wave between Weeks 13 and 20 (range: Weeks 13–20) and a major wave between Weeks 37 and 52 (range: Weeks 42–45). These waves lasted 7 and 15 weeks, respectively. The following intensity thresholds were obtained for the major wave 56.4% for moderate intensity, 69.2% for high intensity and 74.9% for extraordinary intensity. The intensity thresholds for the minor wave were as follows: 36.7% for moderate, 47.7% for high and 52.5% for extraordinary levels of intensity. These thresholds resulted in an overall assessment of each seasonal intensity, which was moderate to high for the 2017–2019 influenza seasons and low to moderate for the 2020–2022 influenza seasons.

This average curve, generated using historical data, was then compared with the different influenza seasons during the pandemic. The 2020 season was the least intense, as no waves were observed during that year: More than 90% of the data were below the seasonal threshold. In the 2021 season, the expected minor wave started earlier in Week 3 and lasted 7 weeks, and the intensity level was high reaching a peak of 52.9%, followed by a major wave starting in Week 35 but not following the normal distribution. Meanwhile, in the 2022 season, the minor wave was observed later between Weeks 18 and 23, and no major wave was identified in the second half of the year, but discrete peaks were observed.

## 4 Discussion

This manuscript compares influenza data before and during the COVID‐19 pandemic and describes SARS‐CoV‐2 data during the period of co‐circulation of both respiratory viruses in Cameroon. Throughout the study period (2017–2022), 24.4% of participants were positive for influenza virus and 2.5% were positive for SARS‐CoV‐2. A recent study by Moumbeket et al. in Cameroon found almost equal circulation of influenza virus and SARS‐CoV‐2 (12.6% vs. 12.8%) during the pandemic period [11]. The reason for the higher SARS‐CoV‐2 detection rate in the latter study is due to the mixed target population, which consisted of persons captured by the influenza sentinel surveillance and from facilities testing suspected cases of COVID‐19. Meanwhile, the lower influenza virus incidence can be attributed to the study period (2020/2021), which is consistent with the decreased global trend in influenza virus observed in many settings during the first year of the pandemic, including Europe, the Americas, Africa, Australia and China [12, 13, 14, 15]. For example, in northern China, the detection rate of influenza decreased dramatically from 13.0% in the 2019/2020 season to 1.4% in the 2020/2021 season [14].

The association between socio‐demographic data and influenza virus infection showed that significantly higher influenza positivity rates were observed in the 2–4‐ and 5–14‐year age groups, representing the school age, in both pre‐pandemic and pandemic periods, as reported in a previous study from Cameroon [8]. In contrast, for SARS‐CoV‐2, all age groups above 15 years were associated with an increased frequency of infection, suggesting that the adult population is more susceptible to this virus, as described in a study from India [16]. Regarding the regional distribution of influenza, higher positivity rates were observed in the forest zones dominated by the littoral and centre regions, similar to the observations of Njouom et al. in 2019 [8]. On the other hand, regions located in the western highlands had higher proportions of SARS‐CoV‐2 infections, although this was supported by a significant association for only one of the two regions. Lower temperatures in these two regions, between 15°C and 19°C [17], may have favoured SARS‐CoV‐2 virus transmission. Several studies describe a negative correlation between the average temperature and the number of SARS‐CoV‐2 infections [18, 19]. However, more data are needed along with climatic and/or socio‐behavioural factors to better explain these trends.

Most of the influenza and SARS‐CoV‐2 cases in this study were mild infections. Some authors have suggested reasons for the relatively low rates of severe disease with SARS‐CoV‐2 infection in Africa, including a high proportion of young people, tropical climate, high physical activity and more time spent outdoors [20]. The data from this study do not support any of these hypotheses, but as our population was predominantly young, this may explain the low rates of SARS‐CoV‐2 in our study. In terms of clinical presentation, myalgia, arthralgia, sore throat, headache, asthenia and rhinorrhoea were found to be significant predictors of influenza infection during the pre‐pandemic period. However, most symptoms were negative predictors of influenza during the pandemic period, including sore throat, headache and asthenia although the exact reason for this change is not known. A previous study from Cameroon identified myalgia, rhinorrhoea and headache as predictors of influenza in Cameroon [21]. One systematic review found that people with influenza had cough, fever, chills and myalgia [22], whereas another found fever, cough and nasal congestion instead [23]. For SARS‐CoV‐2, we identified only sore throat as a positive predictor of infection. Contrary to our findings, other studies found more symptoms associated with SARS‐CoV‐2 infection, including fever, cough, headache, loss of smell or taste and myalgia [24, 25]. The small number of SARS‐CoV‐2 cases detected in our study may have contributed to the poor association of cases with clinical manifestations.

The distribution of influenza subtypes in Cameroon was similar in the pre‐pandemic and pandemic periods, dominated by A(H3N2) subtype. In China, the pre‐pandemic period was dominated by influenza A, and the post‐pandemic period by the B/Victoria lineage [14]. We also noted the disappearance of the B/Yamagata lineage during the pandemic, as confirmed by global influenza statistics [14, 26]. It is not known with certainty what may have contributed to the extinction of this lineage, but this change opens the possibility of switching from quadrivalent to trivalent vaccine, thus increasing production capacity and improving the efficacy of the influenza vaccine [26, 27].

In addition, we recorded a single case of co‐infection with influenza and SARS‐CoV‐2, which presented with mild illness. As influenza and SARS‐CoV‐2 have similar modes of transmission and were circulating at the same time, the likelihood of co‐infection with both viruses was expected to be higher. Other studies from around the world have reported higher proportions of influenza and SARS‐CoV‐2 co‐infection with increased odds of severe illness or death [28, 29, 30, 31]. The fact that the co‐infection was with the influenza B type may be a reason for the milder disease, as some studies have found that co‐infection with influenza A is associated with more severe infection [32, 33]. More research is needed to understand the possible reasons for the low incidence of co‐infection with both viruses in Cameroon.

We observed a change in the seasonality of influenza virus before the COVID‐19 pandemic compared with the pandemic period. The previous reports from Cameroon have confirmed bimodal influenza epidemics: a major wave in the months of September to December and a minor wave in the months of April to June [8, 34]. During the pandemic, three different patterns of influenza seasonality were observed: The epidemic threshold was not reached during the entire 2020 season, whereas in 2021 and 2022, the onset of the two waves shifted from the expected period, with the intensity of influenza activity being higher or lower than expected. A similar observation was reported by Akhtar et al. in Bangladesh, who found that the 2020 influenza season started 18 weeks later, was 7 weeks shorter and less intense than the previous 4 years [35]. This is likely because SARS‐CoV‐2 testing, which was of greater public health importance, diverted all human and material resources dedicated to influenza surveillance, thereby reducing influenza testing and detection rates in 2020. In addition, the implementation of public health and social measures by the Cameroonian government to prevent the spread of SARS‐CoV‐2, such as the closure of educational and training facilities, the prohibition of gatherings of more than 50 people, international travel restrictions, the closure of entertainment venues, the use of face masks and hygiene measures [36, 37], had a direct impact on reducing the frequency of influenza virus detection during the first year of the pandemic. As these measures were relaxed as the pandemic toll declined, the prevalence of influenza and SARS‐CoV‐2 increased again in 2021 and 2022 [37].

A major limitation of this study is the drastic reduction in the number of samples tested in the 2020 season compared to previous years, as this does not allow for an understanding of the true seasonality of influenza during this period. Also, retrospective testing for SARS‐CoV‐2 in 2020 and the first half of 2021 may have contributed to the relatively low detection rates of SARS‐CoV‐2 in these years, as testing was performed on samples that had been repeatedly frozen and thawed. Finally, sample collection from all regions was not equally represented, which biases the comparison of data.

## 5 Conclusion

In conclusion, the COVID‐19 pandemic had a significant impact on the influenza surveillance system, changing its seasonality and reducing its detection rates due to fragile health systems and the imposition of public health containment measures. The vast majority of viruses detected during the pandemic period were influenza viruses, and only one case of double influenza with influenza and SARS‐CoV‐2 was identified. The age distribution of influenza virus, which mainly affects the school‐age population and of SARS‐CoV‐2, which mainly affects the young adult population, suggests that targeted public health measures (such as vaccination) could be implemented to reduce the burden of these infections in Cameroon. The integrated surveillance system for influenza and SARS‐CoV‐2 in Cameroon has been successful in monitoring the circulation of two viruses of public health importance. As the COVID‐19 pandemic has now been declared over as a global health emergency [38], the continuation of this integrated surveillance system is probably the best way to remain vigilant for future respiratory outbreaks.

## Author Contributions

**Gwladys Chavely Monamele:** supervision, validation, writing–original draft, writing–review and editing. **Desmon Toutou Tsafack:** data curation, investigation, writing–review and editing. **Chanceline Ndongo Bilounga:** supervision, validation, writing–review and editing. **Mohamadou Njankouo Ripa:** data curation, investigation, writing–review and editing. **Christian Nsangou Yogne:** data curation, investigation, writing–review and editing. **Hermann Landry Munshili Njifon:** data curation, investigation, writing–review and editing. **Felix Nkom:** supervision, validation, writing–review and editing. **Ubald Tamoufe:** supervision, validation, writing–review and editing. **Linda Esso:** supervision, validation, writing–review and editing. **Fancioli Koro Koro:** supervision, validation, writing–review and editing. **Ronald Perraut:** supervision, validation, writing–review and editing. **Richard Njouom:** conceptualization, supervision, writing–review and editing.

## Conflicts of Interest

The authors declare no conflicts of interest.

### Peer Review

The peer review history for this article is available at https://www.webofscience.com/api/gateway/wos/peer‐review/10.1111/irv.13313.

## Data Availability

The authors confirm that the data supporting the findings of this study are available within the article.
